# Evaluation of Arachnoid Granulations in Cranial Dural Sinuses with Contrast-Enhanced 3-Dimensional T1-Weighted Magnetic Resonance Imaging

**DOI:** 10.5152/eurasianjmed.2023.22104

**Published:** 2023-06-01

**Authors:** Veysel Kaplanoğlu, Hatice Kaplanoğlu, Aynur Turan, Alper Dilli

**Affiliations:** 1 Department of Radiology, University of Health Sciences, Atatürk Sanatory Training and Research Hospital, Ankara, Turkey; 2 Department of Radiology, Health Sciences University Dışkapı Yıldırım Beyazıt Training and Research Hospital, Ankara, Turkiye

**Keywords:** Arachnoid granulations, dural sinuses, magnetic resonance imaging, 3D T1-weighted

## Abstract

**Objective:**

Several studies in the literature have used contrast-enhanced magnetic resonance imaging to investigate arachnoid granulations protruding into the cranial dural sinuses. The current study aimed to investigate the protrusion of arachnoid granulations into the superior sagittal sinus, transverse sinus, straight sinus, and confluence of sinuses and determine the frequency of brain herniation into giant arachnoid granulations using contrast-enhanced 3-dimensional T_1_-weighted magnetic resonance imaging.

**Materials and Methods:**

Images of 550 patients with intra-sinus arachnoid granulations who underwent contrast-enhanced 3-dimensional T1-weighted thin-slice magnetic resonance imaging were retrospectively re-evaluated. Only 300 patients with at least 1 intra-sinus arachnoid granulation were included in the study. The protrusion of arachnoid granulations into superior sagittal sinus, transverse sinus, straight sinus, and confluence of sinuses was investigated. In addition, large arachnoid granulations and brain herniations into arachnoid granulations were also noted.

**Results:**

A total of 889 focal filling defects of arachnoid granulations, at least 1 in the dural sinus, were detected. Of the filling defects of arachnoid granulations, 183 were in the right transverse sinus, 222 in the left transverse sinus, 265 in superior sagittal sinus, 185 in straight sinus, and 34 in confluence of sinuses. Brain herniation into arachnoid granulations was detected in 8 (2.7%) of the patients included in the study. All the filling defects detected in the dural sinuses on post-contrast 3-dimensional T1-weighted images were isointense with cerebrospinal fluid and had round, oval, or lobulated contours. A positive weak correlation was found between patient age and the size and number of arachnoid granulations (*r* = 0.181, *P* < .01 and *r* = 0.207, *P* < .001, respectively). It was observed that the size and number of arachnoid granulations increased as the age of the patients increased.

**Conclusions:**

The distribution, shape, number, and size of intra-sinus arachnoid granulations can vary considerably. Brain herniation into arachnoid granulation can also be seen. Three-dimensional cranial magnetic resonance imaging sequences can be safely used in the evaluation of arachnoid granulations.

**Main Points**

- Intra-sinus arachnoid granulations (AGs) can be found in all intra-cranial sinuses, especially in transverse sinus.
- Brain herniation into AGs can also be seen, and AGs may show inconsistent signals with cerebrospinal fluid.
- As the age of the patients increased, the size and number of AGs also increased.

## Introduction

Intra-sinus arachnoid granulations (AGs) are pseudopodial anatomical structures that protrude into the lumen of the venous sinuses. These structures are filled with cerebrospinal fluid (CSF) and are surrounded by pia-arachnoid membranes. Arachnoid granulations are typically a few millimeters in diameter, but giant AGs larger than 1 cm have also been described.^1^ Most AGs are asymptomatic; however, rarely, they may also be ectatic, requiring differentiation from sinus thrombosis, meningioma, cavernous hemangioma, and meningocele.^2,3^ Giant AGs can obliterate the venous sinuses or be present with the scalloping of the inner table of the calvarium.^4,5^

Typically, AGs are structures that have the same properties as CSF in terms of density or signal intensity, and they are visualized as structures protruding into the venous sinus lumen in routine computed tomography (CT) and magnetic resonance imaging (MRI).^6^ This retrospective study aimed to determine the frequency of AGs protruding into the superior sagittal sinus (SSS), transverse sinus (TS), straight sinus (StS), and confluence of sinuses (ConfS) and determine the frequency and nature of brain herniations into AGs using contrast-enhanced 3-dimensional (3D) T_1_-weighted MRI.

## Materials and Methods

This study was performed according to the ethical standards of the institutional review board. The examinations of the cases who underwent standard contrast-enhanced brain MRI for any reason were retrospectively and randomly evaluated, using the hospital’s picture archiving and communication systems. Five hundred fifty patients who underwent a cranial MRI examination in the radiology clinic between January 2020 and June 2021 and had post-enhanced 3D T1 sequence images were included in the study.

**Figure 1.**
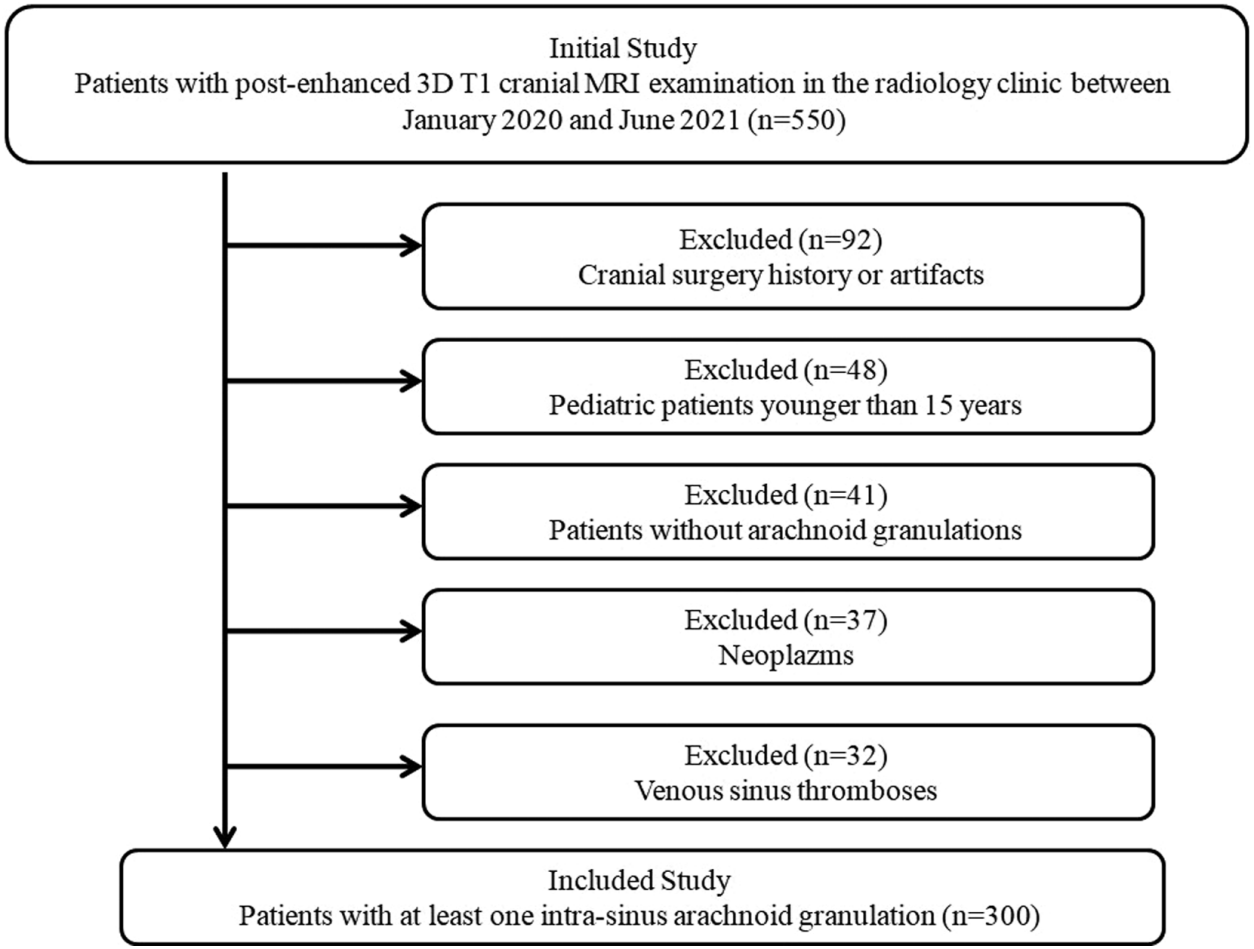
Flowchart shows the numbers of the patients enrolled in the study with inclusion criteria and reduced numbers with exclusion criteria.

All the cranial MRI examinations of 550 patients in the study were re-evaluated by a neuroradiologist with 10 years of experience. In the 3D contrast-enhanced examination, AGs were visualized as well-circumscribed, round or oval-shaped intra-sinus masses, or nodules associated with or adjacent to the cerebral sulcus and cistern, which were homogenous and hypointense on T1-weighted images.^3^ Protrusion of AGs into SSS, TS, StS, and ConfS was investigated. In addition, large AGs and brain herniations into AGs were also noted. When evaluating AGs and brain herniations into AGs, we determined parameters including size, dural venous sinus in which it was located, shape (oval/round), signal characteristics, and presence of contrast-enhancement, which could help exclude other pathologies in the differential diagnosis. Only 300 patients with at least 1 intra-sinus AG were included in the study. Two hundred fifty patients with dural venous sinus thromboses, neoplasms, cranial surgery history or artifacts preventing the visualization of the sinuses, pediatric patients younger than 15 years, and patients without AG in the sinuses were excluded from the study (Figure 1). The basic demographic data (age, gender) of the patients and complaints at the time of presentation to the hospital were screened through the hospital information system.

All the participants were examined in the supine position with a 20-channel head coil using the 1.5 T (Philips Medical Systems, Eindhoven, Netherlands) MRI system. Standard MRI protocols with and without contrast material injection included axial T1-weighted, axial and coronal T2-weighted, axial and sagittal T2-weighted turbo inversion recovery magnitude dark-fluid, and axial Echo-planar imaging (EPI) diffusion sequences. After intravenous gadolinium infusion (Gadoterate meglumine, Dotarem, Guerbet Roissy, France) (0.1 mmol/kg) was administered to the patients, axial, reformatted, coronal, and sagittal 3D T1-weighted MRI images covering the entire cranium were obtained. The imaging parameters of the 3D T1-weighted sequence were as follows: repetition time, 7.3 ms; echo time, 3.5 ms; slice thickness, 1 mm; interslice gap, 0 mm; matrix, 256 × 232; field of view, 256 mm; flip angle, 8°; and scan duration, 3 minutes 36 seconds.

### Statistical Analysis

Mean, standard deviation, median, minimum, and maximum values were used as descriptive statistics for continuous data, and number and percentage values were given for discrete data. The Chi-square test was used for the comparison of AG distributions between the age groups (cross-tables). The relationship between age and size was analyzed using Kruskal–Wallis multiple analysis. International Business Machines’ Statistical Package for the Social Sciences Statistics v. 20 was used in statistical analyses (IBM SPSS Corp., Armonk, NY, USA), and *P* < .05 was accepted as the statistical significance limit.

## Results

The study population consisted of 300 patients, 113 men and 187 women, aged 44.99 ± 15.81 years (15-84 years). The complaints of the patients at the time of presentation to the hospital (headache, dizziness, blurred vision, blurred consciousness, diplopia, vision loss, syncope, vertigo, tinnitus, etc.) were determined, and the most common complaint was headache.

**Figure 2.**
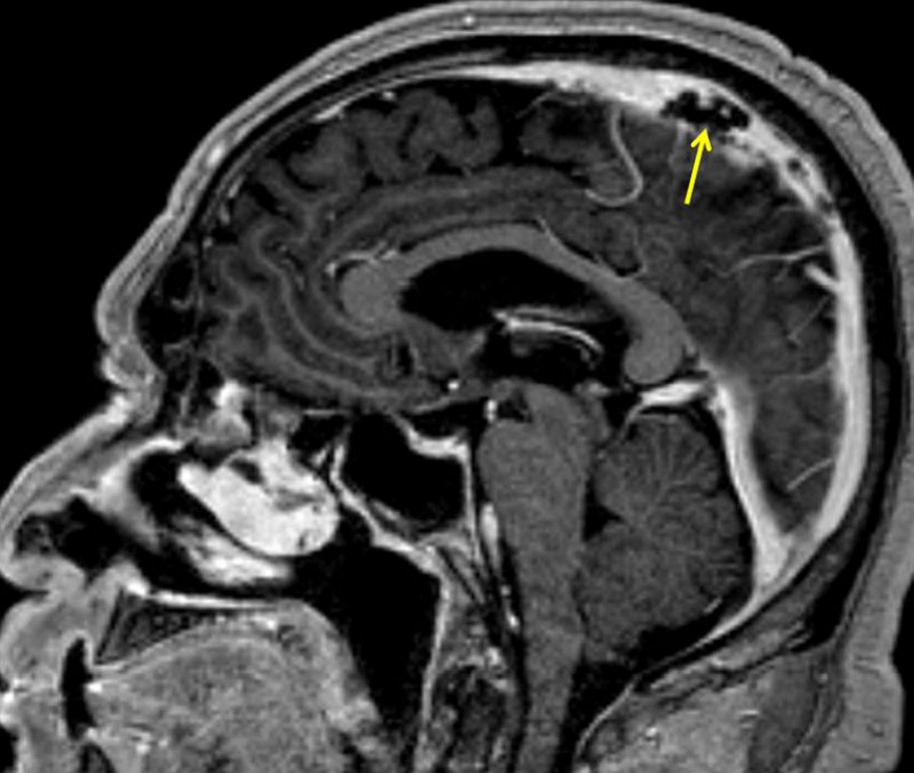
An intra-sinus giant arachnoid granulation in the anterior superior of the superior sagittal sinus (yellow arrow).

Using the post-contrast 3D T1-weighted MRI images, a total of 889 focal filling defects of AGs were detected in at least 1 dural sinus in each of the 300 patients. Concerning the distribution of the number of AGs, there was 1 AG in 23% of the patients, 2 AGs in 24.2%, and 8 AGs in 0.3%. The AGs identified in this study were highly variable in shape, size, and number of the filling defects of AGs, 183 were in the right TS, 222 in the left TS, 265 in SSS (Figure 2), 185 in StS, and 34 in ConfS. According to their localization, AGs were most common in the anterior superior portion of SSS, at the junction with the vein of Galen and in the lower 1/3 of StS, and in the midlateral portion of TS (Table 1). All the filling defects detected in the dural sinuses on the post-contrast 3D T1-weighted images were hypointense compared to the brain parenchyma, isointense with CSF, and had round, oval, or lobulated contours.

**Figure 3.**
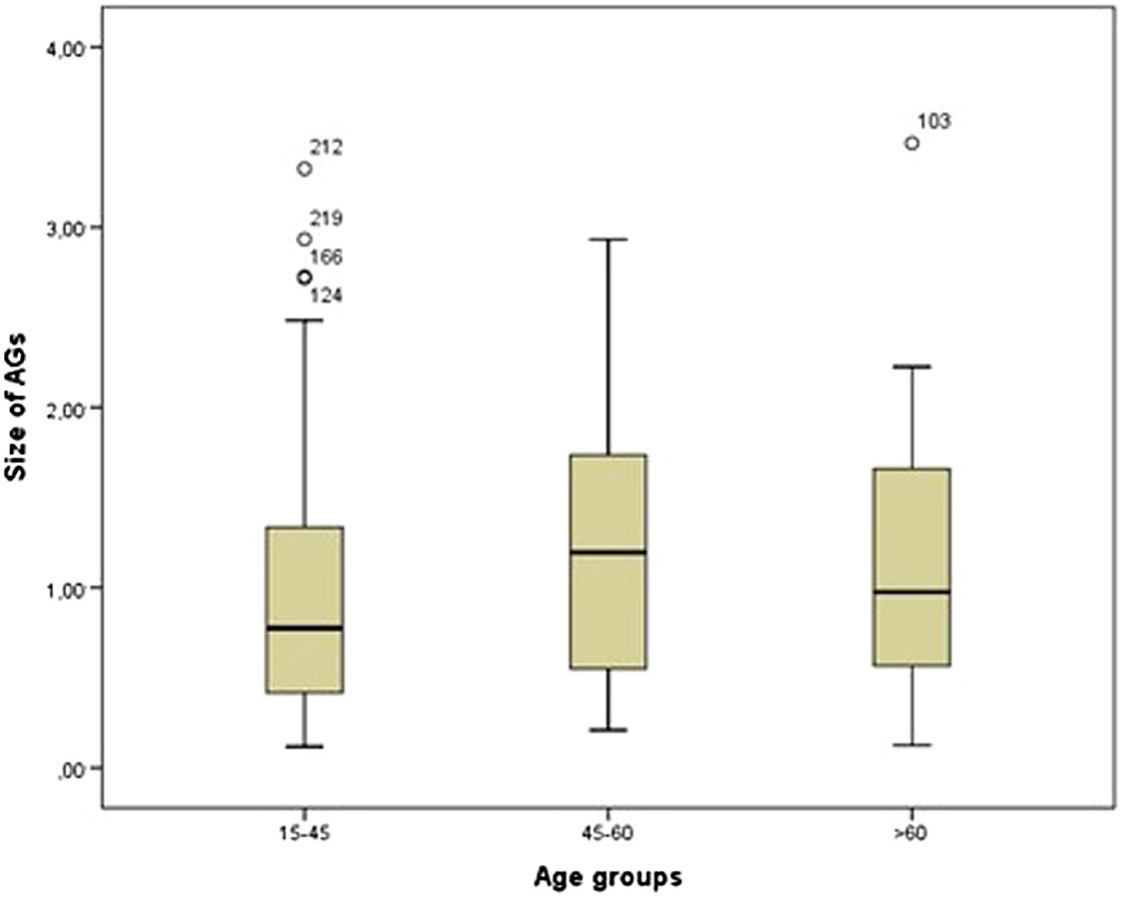
Comparison of the size of AGs according to age groups in the box plot graph. In plot charts, we draw a box from the first quarter to the third quarter. In the median is the line that goes through the box. Lengths go from each quarter to a minimum or maximum. AGs, arachnoid granulations.

**Figure 4.**
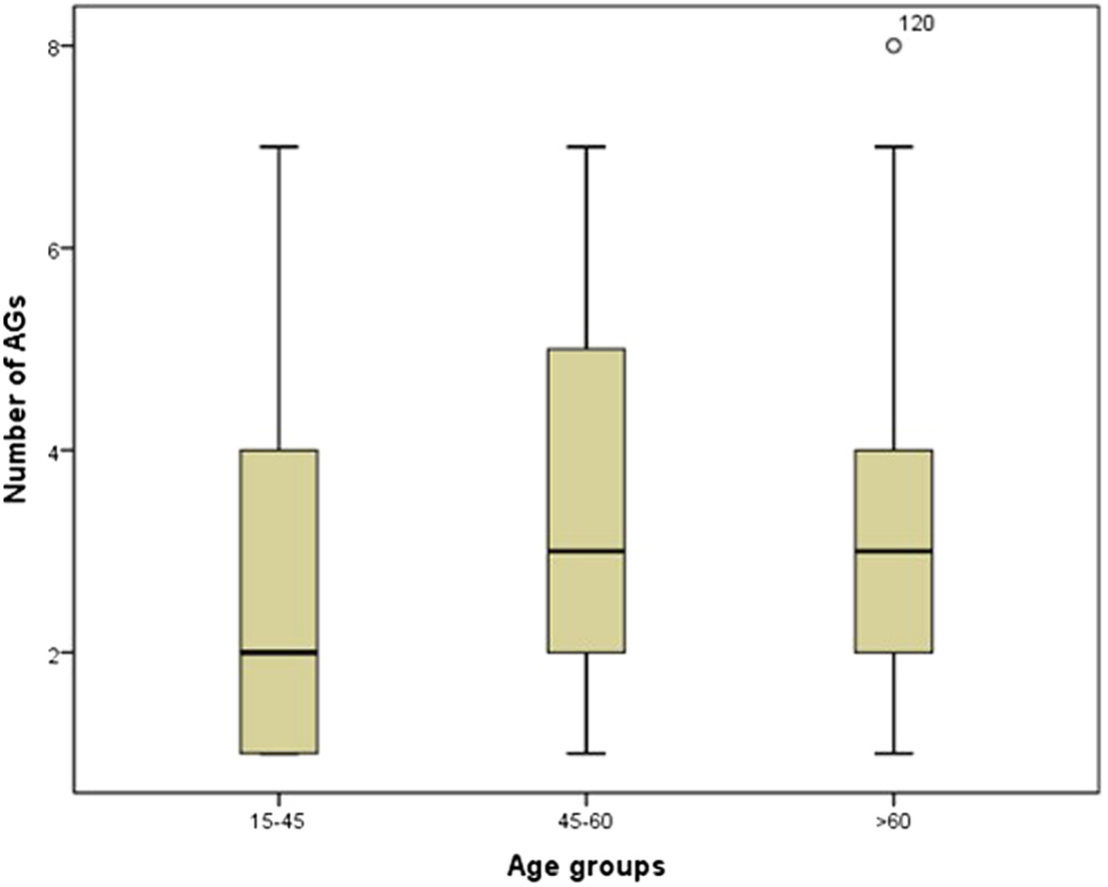
Comparison of the number of AGs according to the age groups in the box plot graph. AGs, arachnoid granulations

The mean AG size was 4.10 ± 2.54 mm (0.7-20 mm). According to their localization, the diameters of AGs were determined as 4.14 ± 2.57 mm for the right TS, 3.85 ± 1.75 mm for the left TS; 4.46 ± 3.19 mm for SSS, 3.85 ± 2.21 mm for StS, and 4.64 ± 1.96 mm for ConfS. There was no difference in arachnoid granulation dimensions between localizations (*P* = .131). There was a difference between the age groups in terms of the AG size (*P* = .022). The AG size was found to be lower in patients aged 15-45 years compared to patients aged 45-60 years (Table 2, Figure 3). The number of AGs was found to be lower in patients aged 15-45 years compared to the 45-60 years group (Figure 4). A positive weak correlation was found between patient age and the size and number of AGs (*r* = 0.181, *P* < .01 and *r* = 0.207, *P* < .001, respectively). As the age of the patients increased, the size and number of AGs also increased.

**Figure 5.**
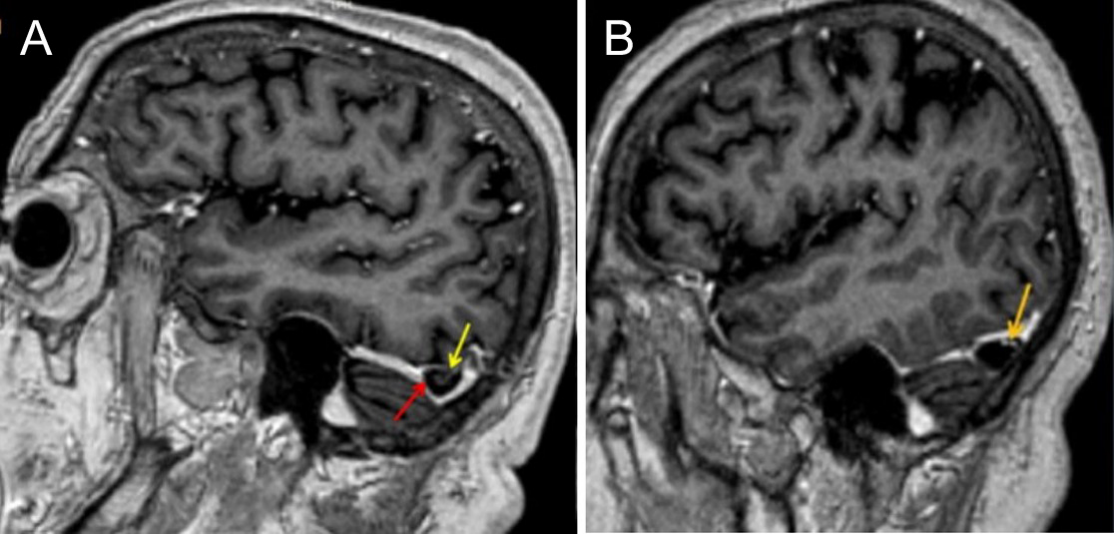
A, B. Sagittal post-contrast T1-weighted MR images show brain herniation (yellow arrow) into an intra-sinus giant arachnoid granulation (red arrow) in the midlateral of the right transverse sinus. MR, magnetic resonance.

Brain herniations into AGs were detected in 8 (2.7%) of the patients included in the study (Figure 5a, b). Around 4 of the brain herniations were into ConfS, 2 were into the midlateral of the right TS, 1 was into the mid portion of the right TS, and 1 was into the left medial TS (Figures 5a, b). The mean size of the brain herniations into AGs was 4.93 ± 1.38 mm. The largest brain herniation was observed in ConfS, measuring 5.52 ± 1.73 mm. Signal characteristics not consistent with CSF were detected in 8 of the patients with brain herniations into AGs. Five cases were isointense with the brain parenchyma, 3 were mildly hypointense, and the remaining were hyperintense with reference to CSF. In 6 cases, brain parenchymal herniation and vascular structure were observed in AGs.

## Discussion

In the evaluation of intra-sinus AGs, 3D high-resolution sequences provide more reliable results. Since 3D images are sequences with a 1-mm section thickness, high resolution, and multiplanar reconstruction capability, they allow for an accurate and reliable evaluation of AGs. Small AGs can be overlooked on standard MRI sequences due to the difficulty of distinguishing between small AGs and the patency of the cortical and bridging veins that connect to the sinuses.^3,4,7^ In the current study, the post-contrast 3D T1-weighted sequences were used for this evaluation.

The AGs detected in the current study were highly variable in terms of shape, size, number, and location. There was 1 AG in 23% of the patients and the rest had more than 1. Two AGs were found most frequently in 24% of the patients. The most common location was TS (45.4%), followed by SSS, StS, and ConfS in the order of frequency. In a study by Tsutsumi et al.^3^ AGs were most commonly detected in TS, followed by SSS, which is similar to our study.

We found AGs to be most commonly located in the anterior superior of SSS, at the junction of the vein of Galen and in the distal 1/3 of StS, and mid-lateral portion of TS, which is consistent with previous studies. Tsutsumi et al^3^ reported that AGs were frequently located in the area close to the transverse-sigmoid sinus junction. In another study using CT and MRI, Leach et al^6^ mostly detected AGs in TS and noted that their localization was associated with venous access, especially in the vein of Labbe. It has been suggested that the frequent occurrence of AGs at the venous junctions is related to the protrusion of the leptomeninges into the areas where the venous structures pass through the dura at the openings to the sinuses.^8^

Koshikawa et al^9^ reported no significant correlation between patient age and the number and size of AGs. In addition, Tsutsumi et al^3^ showed that the mean ages of the patients with and without intra-sinus AGs were similar. In contrast, Haybaeck et al^10^ determined that the frequency of AGs increased with age and Leach et al^6^ suggested that patients with AGs were older than those without AGs. In the current study, there was a positive correlation between patient age and the size and number of AGs. Accordingly, the size and number of AGs increased as the age of the patients increased.

In the literature, Haroun et al^7^ reported that AGs had intermediate signal intensity in one-third of the patients and Leach et al^6^ determined that AGs were hypointense in relation to the brain parenchyma in two-thirds of the patients in the Fluid attenuated inversion recovery (FLAIR) sequence and showed insufficient suppression with reference to CSF in the remaining cases. The authors attributed the occurrence of signal inconsistent with CSF in conventional MRI sequences to the partial volume effect, pulsation effect, and varying CSF flow motion characteristics.^6^ In another study evaluating 45 patients with giant AGs, Oğul et al showed that AGs had an inconsistent signal with CSF in 38 patients. They detected a signal void phenomenon in the AGs of 28 of these patients and brain herniation into AGs in the remaining 10 and argued that the presence of an inconsistent signal with CSF in AGs in conventional MRI may be due to turbulent or jet CSF flow into these structures.^4^ In the current study, the signal of AGs was inconsistent with CSF in patients with brain herniation into AGs. All the cases were isointense with the brain parenchyma and hyperintense with reference to CSF. In 6 cases, brain parenchymal herniation and vascular structure were observed in AGs.

Liebo et al^11^ suggested that brain herniation into AGs may occur secondary to an increase in intracranial pressure or spontaneously. According to Battal et al.^12^ the frequency of brain herniation into AGs in the calvarial or dural sinuses was 0.32%. Oğul et al^4^ evaluated 45 giant AG cases extending to the dural sinus and detected brain herniation in 10 (22%). In our study, a higher rate of brain herniation (2.7%) was found compared to the study of Battal et al, but this rate was even higher in the study by Oğul et al. According to Oğul et al, this is considered to be due to the evaluation of giant AGs. Increasing AG size may increase the rate of intracranial herniation. In addition, Oğul et al used the 3D MRI system and obtained the 3D T1 MPRAGE and 3D SPACE sequences together, which may have facilitated the detection of herniation.

In the current study, the mean AG size was found to be 4.10 ± 2.54 mm (min 0.7-max 20 mm). There was no significant difference in the size of AGs located in different sinuses. In previous studies, the mean size of intra-sinus AGs has been reported to range from 2 to 8 mm. The form exceeding 1 cm and defined as a giant AG is rarely seen.^13^ The strong side of our study is the large number of AG populations included in the study. There are some limitations to our study, the first of which concerns is its retrospective nature. The inhomogeneity of the patient groups in terms of age and the inclusion of only cases with intra-sinus AGs can be considered as other limitations of our study.

In conclusion, intra-sinus AGs can be found in all intra-cranial sinuses, especially in TS. The distribution, shape, number, and size of AGs can vary considerably. As the age of the patients increased, the size and number of AGs also increased. Brain herniation into AGs can also be seen, and AGs may show inconsistent signals with CSF, especially in cases with brain herniation. Knowing these imaging features of AGs can help distinguish especially benign giant AGs from other venous sinus pathologies.

## Figures and Tables

**Table 1.** Distribution of AGs in the Venous Sinuses on Post-Contrast 3D T1-Weighted Images

AG localization	n	%
**Right transverse sinus**	**183**	**20.5**
Right transverse sigmoid junction	31	3.48
Right midlateral transverse	84	9.44
Right medial transverse near torcula	68	7.65
**Left transverse sinus**	**222**	**24.9**
Left transverse sigmoid junction	47	5.3
Left midlateral transverse	99	11.1
Left medial transverse near torcula	76	8.5
**Superior sagittal sinus**	**265**	**29.8**
Anterior and superior portion	170	19.1
Posterior portion	95	10.7
**Straight sinus**	**185 **	**20.8**
Superior portion	37	4.2
Inferior third portion	68	7.6
Junction of the vein of Galen	80	9
**Confluence of sinuses**	**34 **	**3.8**

AG, arachnoid granulation; 3D, 3 dimensional.

**Table 2.** Comparison of the Size of AGs According to Age Groups

	15-45 years	45-60 years	>60 years	Test statistic	*P*
	Mean ± SD Median (Min-Max)	Mean ± SD Median (Min-Max)	Mean ± SD Median (Min-Max)		
AG size	0.94 ± 0.64 0.77 (0.12-3.32)	1.23 ± 0.78 1.19 (0.21-2.93)	1.09 ± 0.98 0.97 (0.13-3.47)	*χ* ^2^ =7.646*	.022

AG, arachnoid granulation; SD, standard deviation.

*Kruskal–Wallis analysis of variance.
